# Full Viral Genome Sequencing and Phylogenomic Analysis of Feline Herpesvirus Type 1 (FHV-1) in Cheetahs (*Acinonyx jubatus*)

**DOI:** 10.3390/v13112307

**Published:** 2021-11-19

**Authors:** Morgan E. Marino, Melanie A. Mironovich, Nikole E. Ineck, Scott B. Citino, Jessica A. Emerson, David J. Maggs, Lyndon M. Coghill, Edward J. Dubovi, Rachel C. Turner, Renee T. Carter, Andrew C. Lewin

**Affiliations:** 1 Department of Veterinary Clinical Sciences, Louisiana State University School of Veterinary Medicine, Baton Rouge, LA 70803, USA; mmari16@lsu.edu (M.E.M.); mironovich1@lsu.edu (M.A.M.); nineck@lsu.edu (N.E.I.); reneecarter@lsu.edu (R.T.C.); 2 White Oak Conservation, Yulee, FL 32097, USA; scitino@white-oak.org (S.B.C.); jemerson@white-oak.org (J.A.E.); 3 Department of Surgical and Radiological Sciences, University of California Davis School of Veterinary Medicine, Davis, CA 95616, USA; djmaggs@ucdavis.edu; 4 Bioinformatics and Analytics Core, University of Missouri, Columbia, MO 65211, USA; lcoghill@missouri.edu; 5 Department of Population Medicine and Diagnostic Sciences, Cornell University College of Veterinary Sciences, Ithaca, NY 14853, USA; ejd5@cornell.edu; 6 Birmingham Zoo, 2630 Cahaba Road, Birmingham, AL 35223, USA; rturner@birminghamzoo.com

**Keywords:** feline herpesvirus, herpes, Varicellovirus, cheetah, genome sequencing, phylogenomic, vaccines, modified live vaccine, veterinary

## Abstract

Feline herpesvirus type 1 (FHV-1) is endemic in captive cheetahs and sporadically causes devastating disease. Modified live vaccines (MLV), intended for use in domestic cats, are used in some captive cheetah populations and have been anecdotally linked to disease in certain subpopulations. Ten FHV-1 isolates from ten captive cheetahs and one isolate from an MLV used to inoculate four of the host animals were analyzed. Viral DNA was extracted for full-genome sequencing by Illumina MiSeq with viral genomes then used for phylogenomic and recombinational analyses. The FHV-1 shed by vaccinated cheetahs were almost identical to the MLV, with few variants among viral genomes. Eight cheetah FHV-1 isolates and the MLV were grouped in a clade along with FHV-1 isolates from domestic cats in the USA. The remaining two cheetah FHV-1 isolates (unknown host vaccine status) were not associated with a clade. The likely ancestral origin of these two isolates involves recombination events between Australian domestic cat and cheetah FHV-1 isolates. Collectively, these data suggest that the MLV is capable of causing clinical disease and viral shedding in some cheetahs and represents evidence of interspecies transmission of virus between domestic and wild cats.

## 1. Introduction

Wild cheetah (*Acinonyx jubatus*) populations are considered “vulnerable” according to the International Union of Conservation Nature (IUCN) but are quickly approaching “endangered” status as populations continue to decline [1]. There has been a concerted effort to maintain an assurance population of cheetahs in North American zoos and conservation centers to combat dwindling wild populations. Low reproduction rates, adult mortalities, and infectious diseases have been major hindrances to maintaining these captive populations [2]. Feline herpesvirus type 1 (FHV-1) is a contributing factor to mortalities and is endemic in captive cheetah populations [3]. 

While the domestic cat is the dominant host, FHV-1 has been isolated from numerous wild felid species, including captive, semi-captive, and free-ranging cheetahs [2,3,4,5]. FHV-1 can cause a wide array of disease manifestations, similar to those observed in the domestic cat. While most cheetahs experience mild disease, others develop severe ocular, respiratory, and dermatologic clinical signs [6,7]. This can result in a life-long infection and clinical signs of disease due to viral latency and persistent viral shedding [3,7]. Cheetahs are believed to be particularly vulnerable to viral diseases due to genetic monomorphisms at the major histocompatibility complex (MHC), which allow FHV-1 to evade the host immune system [8]. Severe disease can lead to humane euthanasia in some animals, which limits captive population growth [9]. 

FHV-1 is a double-stranded DNA virus with a glycoprotein–lipid envelope that belongs to the genus *Varicellovirus* and the subfamily *Alphaherpesvirinae*. Like other alphaherpeviruses, it is characterized by establishing neural latency within its host after the initial development of infection. Current diagnostic methods include viral isolation, polymerase chain reaction (PCR), and serology. While some treatments have been studied in domestic cats, these are not proven to be effective in other species and are often not practical in wild Felidae [4,9,10]. 

Much of what is known about FHV-1 infections in the cheetah is extrapolated from research in domestic cats; the preventative vaccinations and medical treatments used in cheetahs are intended for use in the domestic cat. FHV-1 is included in the core vaccine recommendations for captive non-domestic felid populations [11]. Both killed-virus and modified live vaccines (MLV) are used to prevent FHV-1 in captive cheetahs. Killed-virus vaccines are recommended during breeding, pregnancy, and infancy when maternal antibodies begin to wane. Killed vaccines can reduce the incidence and severity of disease; however, they do not offer holistic protection from infection or prevent viral shedding [3]. MLV contain live attenuated viral strains and are selectively used as boosters in breeding female cheetahs at some breeding centers [12]. MLV induce a more robust immune response that is longer lasting than killed vaccines and are thought to improve the maternal transfer of anti-FHV-1 antibodies in nursing cubs, but they have been anecdotally linked to disease development when used in certain cheetahs [3,10,13]. 

Since vaccinations are only partially effective and treatments are not always practical, understanding the source of infection in cheetahs is crucial for the implementation of proper prevention and control strategies. Earlier reports utilizing restriction endonuclease digestion suggested that FHV-1 isolates from cheetahs were similar to those from domestic cats, but this approach is insufficient to detect the numerous polymorphisms which occur throughout the entire viral genome [14]. It is therefore unknown whether FHV-1 isolates from domestic cats are significantly different to those found in captive cheetahs. Furthermore, the relationship between herpetic disease in cheetahs and administration of MLV is poorly understood. A previous investigation of four FHV-1-positive captive cheetahs using PCR-based sequencing techniques failed to establish a relationship between the MLV and herpetic disease due to the highly conserved nature of the FHV-1 genome [10]. However, despite a high degree of homogeneity in FHV-1 isolates, phylogenomic analysis using full viral genome data of this virus from domestic cats led to the discovery that clear clades can be delineated according to geographic host location [15]. In the present study, we utilize similar techniques to determine the relationship between MLV, the viral strain shed by cheetahs, and FHV-1 strains obtained from domestic cats. 

Our objective was to utilize full FHV-1 genome data to investigate possible sources of infection in captive cheetahs, and specifically to assess the use of MLV and spread from domestic cat populations as potential sources. Based on prior evidence [10], we hypothesized that the MLV is capable of causing clinical disease in this species, and that unvaccinated cheetahs are susceptible to infection from FHV-1 spread from domestic cat populations.

## 2. Materials and Methods

### 2.1. Cells, Viruses, and Host Animals

Crandell Rees feline kidney cells (CRFK; ATCC, Manassas, VA, USA) were used to prepare viral stocks from clinical samples (nasal, ocular, and pharyngeal swabs) obtained from host animals, as previously described [15]. Diagnostic samples were sent in phenol red cell media and frozen for transportation. Cells were cultured in Dulbecco’s modified Eagle medium (DMEM; Life Technologies, Grand Island, NY, USA) with 10% fetal bovine serum (VWR, Radnor, PA, USA) and 1% penicillin and streptomycin sulfate (Life Technologies, Grand Island, NY, USA) at 37 °C and 5% CO_2_.

The viruses sequenced in this study are shown in Table 1. Ten diagnostic samples collected over a 19-year period (2001–2020) from captive cheetahs with FHV-1 disease (housed in zoos or conservation centers in the USA) and one MLV (Purevax Feline 3, Merial, Inc., Athens, GA, USA) were sequenced in this study. Previously sequenced and analyzed viral isolates were included in the phylogenomic analysis, including 52 domestic cat FHV-1 isolates, 2 FHV-1 MLV isolates, and 1 canine herpesvirus (CHV-1) outgroup [15,16]. All host cheetahs were housed in zoos or conservation centers at the time of sampling. Cheetahs from which isolates MM-2, MM-3, MM-7, MM-9, and MM-10 were collected were housed in the same facility in Florida. MM-2, MM-3, and MM-7 shared an enclosure and were all vaccinated with the Merial MLV 2 months prior to the development of clinical disease. Cheetahs from which isolates MM-4 and MM-8 were collected were both housed in the same zoo in California and tested positive for FHV-1 by PCR in June 2016. Their vaccine status and clinical signs are unknown.

### 2.2. Viral Isolation

Samples were stored at −80 °C prior to viral isolation. A 0.5 mL aliquot of each sample was added to individual T75 culture flasks containing maximally confluent CRFK cells along with 3.5 mL of DMEM containing 2% fetal bovine serum and 1% penicillin and streptomycin before being placed on a rocker to incubate at room temperature for 60 min. An additional 6 mL of DMEM was then added to each plate before being incubated at 37 °C and checked daily for up to 7 days until 100% cytopathic effect (CPE) was observed. The infected cells and media in the flask were then frozen at −80 °C and thawed at room temperature for 3 cycles. Supernatant was removed from the flask and placed in a conical tube and centrifuged at 1000 rpm for 5 min (ThermoScientific Heraeus Megafuge 8R centrifuge). The supernatant was pipetted into another conical tube with care not to disturb the cell pellet before being pipetted into cryotubes for preservation at −80 °C.

### 2.3. Viral DNA Extraction

Viral DNA extracted from the frozen supernatant was prepared using a commercial kit according to the manufacturer’s instructions (PureLink Viral RNA/DNA Mini Kit, Invitrogen, Carlsbad, CA, USA). DNA purity and concentration were assessed using a NanoDrop One Microvolume Spectrophotometer (Thermo Scientific, Waltham, MA, USA). Samples were confirmed to contain FHV-1 DNA using quantitative polymerase chain reaction (qPCR; ABI 7900-2; Waltham, MA, USA) on extracted DNA using thymidine kinase primers, which are specific for FHV-1 (IDT, Coralville, IA, USA).

### 2.4. Sequencing

DNA concentration was verified using the Qubit dsDNA HS Assay Kit (Life Technologies, Grand Island, NY, USA). Samples were diluted to 1 ng DNA in 5 mL solution and libraries were constructed according to the Nextera XT DNA Library Preparation Kit protocol (FC-131-1096; Illumina Inc., San Diego, CA, USA). Libraries were amplified and indexed using Nextera XT Index Kit v2 (Set A; Illumina Inc., San Diego, CA, USA). Quality and quantity of finished libraries were assessed using a Fragment Analyzer Instrument (Advanced Analytical, Ames, IA, USA) and dsDNA HS Assay Kit. Indexed libraries were pooled, and paired-end sequenced using Illumina MiSeq 500 bp (v2) sequencing kit (MS-102-2003). 

### 2.5. Genome Assembly

Reference-based assembly was performed using Geneious Prime ver 2020.2.4 as previously described [17]. In summary, paired-end reads were trimmed using BBDuk adapter/quality trimmer ver 38.84 (right end, Kmer length = 27, maximum substitutions= 1, minimum quality = 20, minimum overlap = 20, minimum length = 20). Trimmed paired-end reads were then assembled to FHV-1 reference sequence C-27 (GenBank accession NC_013590). A consensus sequence was extracted from aligned reads with gaps filled with “N’s”. Genomes were then annotated and submitted to GenBank using Geneious Prime ver 2020.2.4.

### 2.6. Viral Genome Alignment

Viral genomes were aligned as previously described using MAFFT ver 7.450 within Geneious Prime ver 2020.2.4 [17,18]. Default parameters were used for all alignments (scoring matrix of 1PAM/k = 2, gap penalty of 1.53 and offset value of 0.123). Alignments of all whole sequenced FHV-1 genomes (USA, China, Australia) were created, which included cheetah FHV-1 for this study and a CHV-1 outgroup (0194, GenBank Accession NC_030117.1). An additional alignment containing isolates from vaccinated animals (MM-2/3/7/9) and the MLV was also created in a similar manner.

### 2.7. Variant Analysis

Analysis of variants was performed as previously described using the Geneious variant finder within Geneious Prime ver 2020.2.4 [17,18]. Each sequenced isolate was compared to the reference genome (C-27) to identify variants.

### 2.8. Phylogenetic and Recombination Analysis

All phylogenetic and recombinational analyses were performed as previously described, with some modifications as follows [17]. An alignment including all available FHV-1 genomes and the CHV-1 outgroup was analyzed (1000 bootstrap replicates) using ModelFinder [19] via IQ-Tree 2 ver 1.6.12 [20], which described the best-fit model (TVM + F + R7). The resultant maximum likelihood tree was visualized using Splitstree ver 4.16.1 [21]. Recombination analysis was carried out using RDP ver 4.100 [22] on the aligned FHV-1 genomes using a manual boot scan [23], RDP [24], GENECONV [25], MaxChi [26], Chimaera [27], SiScan [28], and 3SEQ [29]. Interclade and intraclade genomic distances were calculated using MEGAX [30] using the gamma distribution model, partial deletion of gaps and 1000 bootstrap replicates.

### 2.9. Sequence Accession Numbers

All sequenced isolates are shown in Table 1 and are available on the GenBank website (https://www.ncbi.nlm.nih.gov/genbank/, accessed on 15 November 2021).

## 3. Results

### 3.1. Sequencing and Genome Assembly

Ten diagnostic samples from cheetahs infected with FHV-1 and one FHV-1 MLV (Purevax Feline 3, Merial, Inc., Athens, GA, USA) were used to create viral isolates which were then sequenced using Illumina MiSeq. Fifty-two previously sequenced FHV-1 isolates [15,16], two additional commercially available MLV [16], and one CHV-1 outgroup [17] were included in the analyses. Both male and female cheetahs are represented in this group, with a median age of 6.5 years (9 months–11 years). All animals included in this study were housed in zoos or conservation centers in Florida, California, Texas, and Missouri. Half of the diagnostic samples (5/10) were from cheetahs housed in Florida, with three of those host animals being housed together in the same enclosure (animals from which isolates MM-2, MM-3, and MM-7 were collected).

Table 2 shows the sequencing details from the ten FHV-1 isolates from cheetahs and the newly sequenced Merial MLV strain. The total number of reads ranged from 1,751,690 (Merial MLV strain) to 2,596,572 (MM-2). The number of mapped reads ranged from 520,743 (MM-10) to 1,050,187 (MM-7). The average mapped read length ranged from 168.5 base pairs (MM-7) to 191.4 (MM-10). Mean coverage across the genomes was high; ranging from 729.8X (MM-10) to 1294.6X (MM-7). GC content was consistent throughout all strains, ranging from 45.1% (MM-6) to 45.9% (Merial, MM-1). Mapped genome length ranged from 136,658 (MM-10) to 137,611 (MM-7).

### 3.2. Variant Analysis

Cheetah FHV-1 isolates were compared to C-27, an FHV-1 isolate obtained from a domestic cat host [15], and the Merial MLV. All variants found in the coding regions of the genomes are summarized in Table 3. When compared to C-27, variants were found to be widely distributed throughout the genome. There were 61 total unique variants found, with 28 variants being synonymous and 33 variants being non-synonymous. The gene with the most unique variants was infected cell polypeptide 4 (ICP4), which activates herpesviral gene expression during infection [31]. ICP4 accounted for 10 unique variants: 6 non-synonymous and 4 synonymous. Two of the variations were found in MM-6 while the remaining eight were found in MM-5. 

Isolates from vaccinated host animals (MM-2, MM-3, MM-7, MM-9) were directly compared to the Merial MLV isolate. There were five detected variants within the coding regions of the isolates, with four being in gene UL48 and one being in gene UL41. The variation noted in all cases was a single-nucleotide polymorphism “Y→T” (Y signifies that the base is either a cytosine or thymine). This conservative approach identifies all possible variants, which is most appropriate in a highly conserved viral genome such as FHV-1. In this case, it is quite possible that all coding regions of MM-2, MM-3, MM-7 and MM-9 were actually identical to the MLV. When percent identities were compared for each of the cheetah FHV-1 isolates and the remaining 64 FHV-1 isolates (cheetah FHV-1, domestic cat FHV-1, MLV), the top 10 most similar isolates were consistently other cheetah FHV-1 isolates or an MLV. Isolates from cheetahs which were vaccinated with Merial MLV were most similar to the vaccine (MM-2, MM-3, MM-7, MM-9), with MM-3 having the highest percent identity to the vaccine (99.08%). Percent identities of all isolates compared to the vaccine strain and reference strain C-27 are shown in Table 4.

### 3.3. Phylogenetic and Recombination Analysis

**Figure 1:**
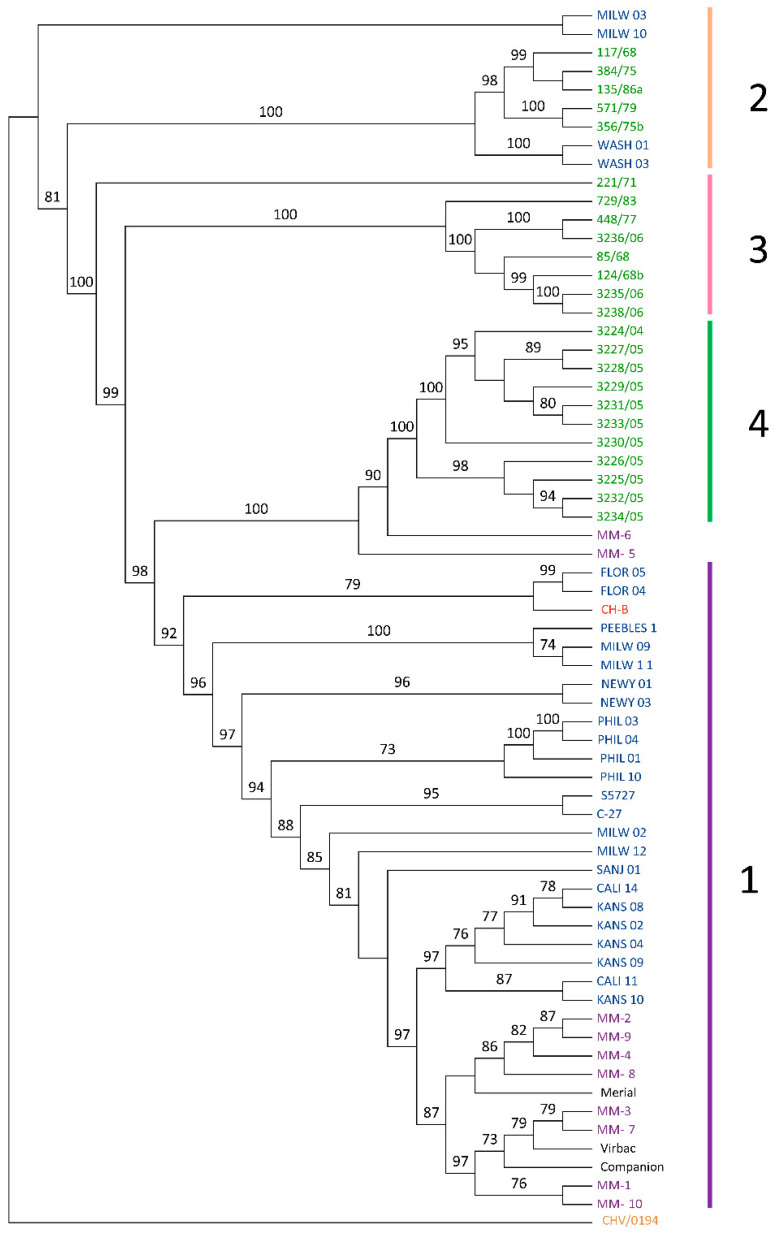
Maximum likelihood tree including all currently available feline herpesvirus type 1 (FHV-1) isolates along with canine herpesvirus (CHV-1/0194) as an outgroup (orange). Eight of ten cheetah isolates (purple) fell into Clade 1, with closest relations to one of the three modified live vaccines (MLV; black). Two cheetah isolates (MM-5 and MM-6) were sisters to FHV-1 strains previously isolated from domestic cats in Australia (green). Those strains in blue are previously analyzed FHV-1 isolates from domestic cats within the USA. CH-B strain (red) was previously isolated from China and falls outside of the clade structure but is closely genetically related to Clade 1.

The best-fit transversional model of the phylogenetic tree (TVM + F + R7) [32] was chosen automatically based on the Bayesian information criterion score (BIC) using ModelFinder [19] within IQTree 2 ver 1.6.12 [20]. The results were used to create a maximum likelihood tree (Figure 1). All bootstrap support values over 70 are shown in the figure. The maximum likelihood tree was visualized using Geneious Prime ver 2020.2.4. 

**Figure 2:**
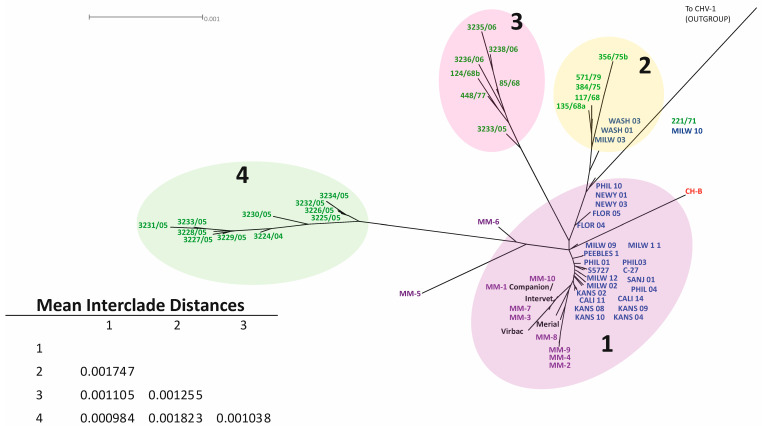
Phylogenetic tree including all currently available FHV-1 sequences with a CHV-1 outgroup. Domestic cat FHV-1 isolates from the USA are shown in blue, one isolate from China is shown in red, isolates from Australia in green, isolates from modified live vaccines (MLV) in black, and cheetah FHV-1 isolates are shown in purple. Four clades are shown: Clade 1 includes only USA isolates (with vaccine strains and cheetah strains), Clade 2 includes both Australian and USA isolates, and Clade 3 and Clade 4 contain only Australian isolates. Mean interclade distances were calculated using MEGAX. MM-2, MM-3, MM-7, and MM-9 were isolated from cheetahs known to be vaccinated with the Merial brand MLV. All 4 of these isolates show a very high degree of similarity to the MLV.

The same data were used to create a phylogenomic network/cladogram using Splitstree ver 4.1.6 [21], which allowed for additional confirmation of clade structure (Figure 2). Note that the original clade designations from an earlier publication were maintained for clarity and consistency [15]. Clade 1 contained previously sequenced domestic cat strains from the US, cheetah strains, and vaccine strains. Eight of the ten cheetah isolates clustered into clade 1, including those with known MLV vaccine status (MM-2, MM-3, MM-7, MM-9). Clades 3 and 4 contained isolates previously sequenced from domestic cats in Australia [16]. Clade 2 comprised previously sequenced isolates from domestic cats within the United States and Australia [15,16]. As hypothesized, isolates obtained from host animals who were vaccinated shared homogeneity with the MLV and were grouped closely together (Figure 2).

The mean overall genomic distance for all sequenced FHV-1 isolates was 0.098%, which is comparable to the previously reported overall mean interisolate distance of 0.093% [15]. Interclade genomic distances ranged from 0.000984 (between clades 1 and 4) to 0.001823 (clades 2 and 4). All interclade genomic distances are reported in Figure 2.

A recombination analysis was performed on an alignment containing all available FHV-1 sequences using manual bootscan, RDP, GENECONV, MaxChi, Chimaera, SiScan, and 3SEQ [22]. There was no evidence of recombination between vaccine isolates and isolates from cheetah or domestic cat hosts. For the cheetahs, this is presumably due to a very high degree of genomic similarity between vaccinal and wild-type viruses. There was evidence that cheetah isolate MM-6 originated from a recombination event between cheetah FHV isolate MM-8, the major parent, and an Australian domestic cat isolate 3229/05 [16], the minor parent. The likely origin of MM-5 was recombination events between two isolates (major parent 729/83; minor parent 117/68) previously obtained from domestic cats within Australia [16]. In both cases, *p* < 0.0001 (unbiased values derived using the unweighted pair group method with arithmetic mean (UPGMA) tree topology test).

## 4. Discussion

This study represents the first full viral genome sequencing and phylogenomic analyses of FHV-1 isolated from captive cheetahs. The isolates sequenced from cheetahs which had received the MLV were highly genetically similar to the strains isolated from MLV. Two FHV-1 isolates from cheetahs were genetically different from other cheetah isolates and were not grouped into a clade: MM-5 (isolated from a male cheetah in Texas in 2013) and MM-6 (isolated from a cheetah in Missouri in 2001). There is suggestive evidence of MM-5 and MM-6 arising from a common ancestor, as they are basally nested in the maximum likelihood tree (Figure 1). MM-5 is more distantly related to FHV-1 isolates from Australian domestic cats; however, there is supportive evidence that it is related to these isolates. While it is likely that these two strains are related, it is also possible that they arose from two independent evolutionary events. Further recombinational analyses suggest that the likely origin of MM-5 was a recombination between two strains (729/83; 117/68) previously isolated from domestic cats within Australia [16]. The likely origin of MM-6 is recombination between a cheetah strain (MM-8) isolated within the USA and a strain previously isolated from a domestic cat in Australia (3229/05) [16]. This finding is supported by previous research suggesting that recombination is highly prevalent in various herpesviruses [33,34,35].

Analysis showed ~99% shared genomic identity between FHV-1 from MLV vaccinated cheetahs and the MLV isolate and ~98% shared genomic identity between isolates from domestic cats and cheetahs. This is a relatively large difference for a highly conserved virus such as FHV-1. Our results do not indicate a 100% similarity between FHV-1 in vaccinated animals and the MLV. There are several possible explanations for this finding. It is likely that limited point mutations can develop in the FHV-1 MLV following administration to live animals over a short period of time, or when cultivated in vitro. Herpesviruses mutate readily in vitro in cell culture conditions, with mutation rates being higher within more diverse viral populations [36]. Since these viral populations were isolated from diagnostic samples and not purified, this is expected to be a likely explanation for the limited variants observed. Of the observed variants in the coding regions, almost all of these were due to a conservative approach in variant detection, with base ambiguity being a likely explanation, rather than true variation. While the FHV-1 genome is relatively homogenous between isolates, individuals can harbor multiple isolates of a single species of virus, of which only one is subsequently sequenced [37]. Though less likely given the consistency of the results presented, we also cannot rule out potential sources of stochastic error (e.g., PCR or library preparation errors and sequencing errors), as despite significant recent advances in accuracy, these are still potential sources of variation between samples [38,39]. The least likely explanation appears to be that FHV-1 from vaccinated cheetahs is coincidently the most similar to the MLV.

It is unclear why the MLV is associated with clinical disease in only a subset of cheetahs which receive this vaccination. In a paper investigating the reversion to virulence of a modified live varicella zoster virus (VZV) vaccine, it was found that reversion to virulence was due to specific alleles within certain individuals, rather than the virus itself [40]. This may also be the case with some cheetahs and the FHV-1 MLV. However, this does not seem particularly likely given the low degree of genomic variation noted to date within captive cheetahs [8]. Another possible explanation is the recombination of different vaccine strains causing reversion to virulence within the host. This has been previously described in poultry flocks vaccinated with different brands of attenuated gallid herpesvirus type 1 [41]. This is quite unlikely in the vaccinated cheetahs in the present report, since detailed vaccine records were kept for these animals and the same MLV was repeatedly used. Several of the host animals in this study were housed together and all received MLV concurrently. It is common for cheetahs to groom one another; in this case, while they were potentially actively shedding FHV-1. The viral load that these animals were exposed to could therefore have been relatively high, with cross-contamination occurring between animals. Finally, it is known that FHV-1 reactivation is driven by physiologic stressors in domestic cats [4]. Certain cheetahs may be particularly susceptible to this factor, which would explain why only a subset of vaccinated animals subsequently develop clinical signs of FHV-1. Co-infection with multiple pathogens increases the severity of clinical signs in domestic cats with feline respiratory disease complex [42]. Further analyses of the diagnostic samples used in this study for Feline Calicivirus, *Mycoplasma felis*, *Bordetella bronchiseptica*, and *Chlamydophilia felis* could assist in determining if comorbid respiratory infections contributed to certain vaccinated cheetahs being more susceptible to FHV-1 than others.

With further evidence that the MLV is capable of causing clinical disease in certain vaccinated cheetahs, additional work to develop mitigation tactics or alternative approaches for this species are essential. While the MLV appears to be effective in most animals, the prediction of those which will go on to develop clinical disease would be extremely useful for the future management of this species.

FHV-1 isolates from two cheetah hosts did not appear to be related to the use of MLV. In both cases, recombination events involving FHV-1 from domestic cat hosts appear to be the most likely origins of these viruses. This highlights the importance of limiting contact between captive cheetahs and domestic cats, where possible. FHV-1 is known to be readily transmitted through aerosolization and can survive on fomites for 18 hours [3,43,44]. Excellent hygiene measures must be in place for those in contact with captive cheetahs to prevent the introduction of infection.

## 5. Conclusions

The results of this study support the anecdotal conjecture that FHV-1 MLV could be associated with herpetic disease in certain cheetahs. The reason for the variable susceptibility among cheetahs is currently unknown. The data also suggest potential horizontal and trans-boundary transmission of FHV-1 between domestic and wild felid species as a source of infection in these populations.

## Figures and Tables

**Table 1:** Demographic and clinical data for feline herpesvirus type 1 (FHV-1) strains isolated from 10 captive cheetahs and the Merial Purevax Feline 3 MLV.

Strain ID	GenBank Accession Number	Host Age (Years)	Host Sex	Host Species	Host Location	Sample Collection Date (Day/Month/Year)	Sample Source	Vaccine Status (Day/Month/Year)	Clinical Signs
MM-1	OL321946	Unknown	Unknown	Cheetah	Unknown	2005	Unknown	Unvaccinated	Unknown
MM-2	OL410287	11	Female	Cheetah	Florida	16/12/2020	Nasal swab	Merial MLV (16/12/2016)	None
MM-3	OL410288	3.5	Female	Cheetah	Florida	16/12/2020	Nasal swab	Merial MLV (20/7/2020)	Chronic dermatitis
MM-4	OL410289	9	Female	Cheetah	California	3/6/2016	Nasal swab	Unknown	Unknown
MM-5	OL410290	6	Male	Cheetah	Texas	13/11/2013	Nasal and pharyngeal swab	Unknown	Unknown
MM-6	OL410291	Unknown	Unknown	Cheetah	Missouri	6/4/2001	Nasal and ocular swab	Unknown	Unknown
MM-7	OL410292	3.5	Female	Cheetah	Florida	16/12/2020	Nasal swab	Merial MLV (20/7/2020)	Sneezing
MM-8	OL410293	9	Female	Cheetah	California	1/6/2016	Ocular swab	Unknown	Unknown
MM-9	OL410294	7	Female	Cheetah	Florida	16/12/2020	Nasal swab	Merial MLV (11/11/2020)	None
MM-10	OL410295	0.75	Male	Cheetah	Florida	26/5/2009	Nasal swab	Unknown	Productive cough and anorexia
Merial MLV	OL410296								

**Table 2:** Details of sequencing output for feline herpesvirus type 1 (FHV-1) strains isolated from 10 captive cheetahs (MM-1 through MM-10) and that in a modified live vaccine (MLV).

Strain	Number Reads	Mapped Reads	Average Mapped Read Length (bp)	Mean Coverage Per Base	GC Content (%)	Mapped Genome Length
MM-1	2,224,892	894,919	183.1	1199	45.9	137,392
MM-2	2,596,572	724,887	183.0	970.2	45.8	137,037
MM-3	2,438,790	790,548	183.6	1060.8	45.7	137,432
MM-4	2,481,272	834,057	191.0	1167.1	45.3	136,996
MM-5	2,200,776	609,757	179.6	801.6	45.2	136,712
MM-6	2,537,734	832,788	190.9	1162.9	45.1	136,761
MM-7	2,507,912	1,050,187	168.5	1294.6	45.7	137,611
MM-8	2,106,004	693,291	188	955.2	45.3	136,818
MM-9	1,983,946	640,857	178.6	836.2	45.8	136,966
MM-10	2,199,852	520,743	191.4	729.8	45.3	136,658
Merial MLV	1,751,690	854,840	179.5	1122.2	45.9	137,390

**Table 3:** Variants detected among feline herpesvirus type 1 (FHV-1) strains isolated from 10 captive cheetahs, a reference FHV-1 strain (C-27), and the viral strain in the Merial modified live vaccine (MLV). Note the infrequent detection of unique synonymous (0) and non-synonymous variants (5) between the MLV strain and isolates from vaccinated cheetahs. Variants were identified using Geneious Prime ver 2020.2.4.

Gene	Number of Unique Variants (FHV-1 All Cheetah Isolates Compared with C-27)		Number of Unique Variants (MLV Strain Compared with Isolates from Vaccinated Cheetahs (MM-2, -3, -7, and -9))	
	Synonymous Variants	Non-Synonymous Variants	Synonymous Variants	Non-Synonymous Variants
*ICP4*	4	6	0	0
*UL10*	0	1	0	0
*UL12*	2	0	0	0
*UL16*	0	2	0	0
*UL17*	0	1	0	0
*UL19*	1	0	0	0
*UL20*	2	0	0	0
*UL25*	1	2	0	0
*UL28*	2	1	0	0
*UL29*	0	1	0	0
*UL32*	1	0	0	0
*UL36*	3	0	0	0
*UL38*	0	2	0	0
*UL4*	1	0	0	0
*UL40*	1	1	0	0
*UL41*	2	3	0	1
*UL43*	1	0	0	0
*UL44*	0	1	0	0
*UL47*	1	0	0	0
*UL48*	0	1	0	4
*UL50*	0	1	0	0
*UL52*	0	1	0	0
*UL53*	1	0	0	0
*UL54*	1	1	0	0
*UL55*	0	1	0	0
*UL7*	0	1	0	0
*UL9*	1	0	0	0
*US2*	1	0	0	0
*US4*	1	0	0	0
*US7*	0	1	0	0
*US9*	0	1	0	0
*UL3.5*	1	1	0	0
*UL35*	0	1	0	0
*UL5*	0	1	0	0
*UL6*	0	1	0	0

**Table 4:** Percent identities between each feline herpesvirus type 1 (FHV-1) strain isolated from 10 captive cheetahs and that in the Merial MLV or the reference FHV-1 isolate (C-27). In all cases, the percent identity was higher for the MLV strain than for the FHV-1 reference isolate.

Isolate	Percent Identity with Merial MLV	Percent Identity with Reference FHV-1 Isolate (C-27)
MM-1	99.03	98.31
MM-2	99.04	98.32
MM-3	99.08	98.38
MM-4	98.88	98.17
MM-5	98.62	98.05
MM-6	98.70	98.23
MM-7	98.86	98.43
MM-8	99.04	98.35
MM-9	99.01	98.39
MM-10	99.05	98.33

## Data Availability

All sequenced isolates are available on the GenBank website (https://www.ncbi.nlm.nih.gov/genbank/, accessed on 15 November 2021).
